# Types of Variants Among Increased Cases of COVID-19 in the Western Region of Saudi Arabia in June 2021

**DOI:** 10.7759/cureus.26016

**Published:** 2022-06-16

**Authors:** Abdulmohsen M Alahmad, Shady A Kamel, Suhaib T Alsulimani, Mohammad S Alharbi, Fawaz R Alyazidi, Yasser A Allhaybi

**Affiliations:** 1 Field Epidemiology Training Program, Saudi Ministry of Health, Riyadh, SAU; 2 Infectious Disease Control Department, Saudi Ministry of Health, Makkah, SAU

**Keywords:** saudi arabia, vaccine, variants, covid-19, sars-cov-2

## Abstract

Background

The western region of Saudi Arabia is the most populous and diverse. This study aimed to identify the types and distribution of the severe acute respiratory syndrome coronavirus 2 (SARS-CoV-2) variants causing cases of coronavirus disease 2019 (COVID-19) in this region in June 2021.

Methods

We conducted a cross-sectional study. All genetically tested COVID-19 patients were included. We investigated the types, distribution, and magnitude of SARS-CoV-2 variants among cases of COVID-19 in June 2021. We gathered patient demographic data, clinical profiles, and epidemiology data.

Results

Of 115 COVID-19 confirmed patients (mean age, 40 years), 56.5% were males and 43.5% were females. Of those vaccinated, 47.1% had received a one-dose vaccination; 52.9% had received two-dose vaccinations, and 23.6% were unvaccinated. Of those vaccinated, 72.1% had received the Pfizer BioNTech vaccine, and 16.5% had received the Oxford-AstraZeneca vaccine. The Delta variant of SARS-CoV-2 was prevalent in most (87.8%) patients. Among those infected, 28.8% reported contact with another COVID-19 case, and 19.8% reported a travel history. Most cases (68.6%) were moderate, 99.4% of patients recovered, and one patient died from COVID-19.

Conclusion

Most of the cases were primary infections, and the Delta variant was predominant and highly transmissible. Most COVID-19 patients were mild to moderately ill. A better understanding of the transmission and diagnosis of these variants will help in early detection and reduction of infection by application of the best preventive measures.

## Introduction

Since the onset of the coronavirus disease 2019 (COVID-19) pandemic, different variants of the causative virus-the severe acute respiratory syndrome coronavirus 2 (SARS-CoV-2)-have been discovered globally, including the Alpha (B.1.1.7), Beta (B.1.351), and Delta (B.1.617.2) variants. The medical community must further understand their transmission and diagnosis to detect them early and reduce infection by applying the best preventive measures [1].

The Delta variant was initially detected in India in September 2020 and subsequently spread to 111 countries by July 2021 [1]. The Delta variant is exceptionally contagious and estimated to be more than twice as infectious as prior variants. Some data suggest that it may cause more severe sickness in unvaccinated individuals than other strains [2]. Early studies showed that fully vaccinated individuals can transmit the Delta variant [3]. In many communities, low vaccination coverage contributed to a rapid and significant increase in cases associated with the Delta variant, which raised the risk of the emergence of additional variants [4].

In Saudi Arabia, the first COVID-19 case was confirmed on March 2, 2020, by a citizen with a travel history from Iran via the Kingdom of Bahrain [5]. As of December 14, 2021, there had been 550,304 confirmed COVID-19 cases in Saudi Arabia, and more than 48 million vaccine doses were administered, including the booster dose, to people aged 12 years and older [6].

The western region of Saudi Arabia has a large population, and many visit the area in different seasons, making this region critical to study for future disease prevention and control. According to a demographic survey done by the Saudi General Authority for Statistics in 2016, 8.3 million people live in the western region of Saudi Arabia [7]. In December 2020, Saudi Arabia approved the Pfizer-BioNTech vaccine and the Oxford-AstraZeneca vaccine. In July 2021, the Johnson and Johnson vaccine and the Moderna vaccine were approved [8]. However, clinical data on the efficacy of these vaccinations against this variant are limited. Therefore, this study aimed to detect the distribution and magnitude of the Delta variant compared to other variants and to determine the demographic characteristics, patterns, and most probable associated factors of the increased cases of COVID-19 in the western region of Saudi Arabia in June 2021.

## Materials and methods

Study design and population

This is a cross-sectional study, conducted in the western region of Saudi Arabia on all confirmed COVID-19 cases in June 2021.

Inclusion criteria

All confirmed COVID-19 cases that were genetically tested to detect the variant type in regional laboratories were included in the study.

Exclusion criteria

Any cases without genome sequencing were excluded.

Data collection

Extracted data were gathered from the COVID-19 records from the western Saudi Arabian region health sector. We recorded COVID-19 patients’ age, gender, nationality, region, occupation, variant type, symptoms, the severity of illness, patient status, vaccination status, vaccine type, and travel history. The severity of symptoms is classified according to Saudi Center for disease prevention and control [9] and WHO classification [10].

Statistical analysis

We used IBM SPSS Statistics for Windows, Version 21.0. (IBM Corp., Armonk, NY) to analyze the data. As appropriate, descriptive data were presented as numbers and percentages or means and standard deviations. For all purposes, statistical significance was determined at p<0.05.

Ethical considerations

The collected data were managed privately, and all patient identifying information was kept anonymous. The ethical committee of the research center at King Fahad Medical City provided approval for the study (IRB Log Number: 22-019E).

## Results

A total of 115 patients with confirmed COVID-19 were included in the study. Table 1 shows the demographic data. The study included 65 male patients (56.5%) and 50 female patients (43.5%). The mean age of the patients was 40.7 years with the range being nine to 95 years. Most patients were aged 19 to 55 years (67%). Most COVID-19 cases were Saudi nationals (87%), and most lived in the western region (87%). Most subjects were employed (64.3%), and household employment was the most common (19.1%).

**Table 1:** Demographic information of all genetically tested COVID-19 cases in the western region of Saudi Arabia in June 2021

Demographic Variable	N (%)
Gender	
Male	65/115 (56.5)
Female	50/115 (43.5)
Age Group	
1-20	11 (9.6)
21-30	31 (27)
31-40	20 (17.3)
41-55	24 (20.9)
>55	29 (25.2)
Nationality	
Saudi	100/115 (87)
Non-Saudi	15/115 (13)
Place of Residence	
Western region	100/115 (87)
Another region	15/115 (13)
Employment	
Unemployed	41/115 (35.7)
Employed	74/115 (64.3)
Employment type	
Household	22/115 (19.1)
Private sector	14/115 (18.9)
Military sector	14/115 (18.9)
Governmental sector	4/115 (5.4)
Student	8/115 (10.8)
Teacher	8/115 (10.8)
Health care worker	4/115 (5.4)

Table 2 shows clinical data regarding the SARS-CoV-2 variants. Genomic sequencing revealed three variants: Alpha (B.1.1.7), Beta (B.1.351), and Delta (B.1.617.2). The Delta variant was the predominant variant in our study (n=101, 87.8%). Seventy-seven patients (67%) of all confirmed cases had symptomatic COVID-19 and most experienced moderate symptoms (68.6%). One hundred eleven patients (99.4%) recovered from the illness, and only one COVID-19 patient (0.6%) died. Most participants were vaccinated (76.4%), while 23.6% were unvaccinated. Most vaccinated individuals had received the Pfizer-BioNTech vaccine (72.1%). Only 36 people (52.9%) had received the second dose of the vaccine. Thirty-four people (29.8%) had confirmed exposure to a COVID-19 case, and 17 people (19.8%) had a travel history.

**Table 2:** COVID-19 clinical data of all genetically tested COVID-19 cases in the western region of Saudi Arabia in June 2021 Abbreviation: COVID-19, coronavirus disease 2019.

Data	N (%)
Variant type	
Alpha (B.1.1.7)	11/115 (9.6)
Beta (B.1.351)	3/115 (2.6)
Delta (B.1.617.2)	101/115 (87.8)
Symptoms	
Yes	77 (67)
No	38 (33)
Severity of Symptoms	
Mild	12 (14)
Moderate	59 (68.6)
Severe	6 (7)
Patient status	
Recovered	114 (99.4)
Died	1 (0.6)
Vaccinated	
Yes	68 (76.4)
No	47 (23.6)
Vaccine type	
Pfizer-BioNTech	49 (72.1)
Oxford-AstraZeneca	19 (16.5)
Vaccine doses	n (%)
First	32 (47.1)
Second	36 (52.9)
Contact with confirmed case	
No	81 (70.2)
Yes	34 (29.8)
Travel history	
No	69 (80.2)
Yes	17 (19.8)

Table 3 shows the relation between the variant type and vaccine. Sixty-three people (62.4%) with the Delta variant received one dose of the vaccine, 46 (73%) had Pfizer-BioNTech, and 17 (27%) had the Oxford-AstraZeneca vaccine. Thirty people (47.6%) had received only one dose, and 33 (52.4%) had received both doses. We found no statistically significant association between variant type and vaccine data.

**Table 3:** Relation between the SARS-CoV-2 variant and vaccine of all genetically tested COVID-19 cases in the western region of Saudi Arabia in June 2021

Vaccine Data	SARS-CoV-2 Variant			Chi-square Test value	P-value
	Alpha, 11/115 (9.6%)	Beta, 3/115 (2.6%)	Delta, 101/115 (87.8%)		
Vaccinated					
No	8 (72.7%)	1 (33.3%)	38 (37.6%)	5.23	0.073
Yes	3 (27.3%)	2 (66.7%)	63 (62.4%)		
Type of Vaccine					
Pfizer-BioNTech	2 (66.7%)	1 (50%)	46 (73%)	0.56	0.757
Oxford-AstraZeneca	1 (33.3%)	1 (50%)	17 (27%)		
Dose					
First	0 (0%)	2 (100%)	30 (47.6%)	4.93	0.085
Second	3 (100%)	0 (0%)	33 (52.4%)		

Table 4 shows the analysis of variant type and travel. Seventy-one patients with the Delta variant (70.3%) had no contact with another confirmed COVID-19 case. Thirteen patients with the Delta variant (16.7%) had a travel history 14 days before the infection was diagnosed. We found a statistically significant association between variant type and travel history (χ2 =6.88, df =2, p = 0.032).

**Table 4:** Relation between variant type and travel status of all genetically tested COVID-19 cases in the western region of Saudi Arabia in June 2021 Abbreviation: COVID-19, coronavirus disease 2019; SARS-CoV-2, severe acute respiratory syndrome coronavirus 2.

	SARS-CoV-2 Variant			Chi-square Test value	P-value
	Alpha, 11/115 (9.6%)	Beta, 3/115 (2.6%)	Delta, 101/115 (87.8%)		
Contact with COVID-19 Case					
No	7 (63.6%)	2 (66.7%)	71 (70.3%)	1.08	0.584
Yes	4 (36.4%)	0	30 (29.7%)		
Travel History					
No	4 (36.4%)	0	65 (64.3%)	6.88	0.032*
Yes	3 (27.3%)	1 (33.3%)	13 (12.9%)		
Contact with travelers					
No	3 (27.3%)	1 (33.3%)	5 (5%)	4.65	0.098
Yes	0	0	8 (8%)		

Table 5 shows the analysis of variant types according to symptoms. Seventy patients (69.3%) with the Delta variant had symptomatic COVID-19, and most of those were moderate (n=55; 78.6%). Only one patient died. We found significant association between variant type and symptoms (χ2 =8.93, df=2, p = 0.011).

**Table 5:** Relation between variant type and COVID-19 symptoms of all genetically tested COVID-19 cases in the western region of Saudi Arabia in June 2021

	SARS-CoV-2 Variant			Chi-square Test value	P-value
	Alpha, 11/115 (9.6%)	Beta, 3/115 (2.6%)	Delta, 101/115 (87.8%)		
COVID-19 Symptoms					
No	8 (72.7%)	2 (66.7%)	31 (30.7%)	8.93	0.011*
Yes	3 (27.3%)	1 (33.3%)	70 (69.3%)		
Severity of Symptoms					
Mild	0	0	10 (14.3%)	1.08	0.898
Moderate	3 (100%)	1 (100%)	55 (78.6%)		
Severe	0	0	5 (7.1%)		
Patient status					
Recovered	7 (63.3%)	1 (33.3%)	77 (76.2%)	0.10	0.949
Died	0	0	1 (0.9%)		

Table 6 shows the analysis of variant types by demographics. Most patients with the Delta variant were male (n=58; 57.4%), and (n=43; 42.6%) were female. Ninety-one Delta cases (90%) were Saudis, most of whom were employed. We found a significant association between the variant detected and nationality (χ2 =7.24, df=2, p = 0.027).

**Table 6:** Relation between variant type and demographics of all genetically tested COVID-19 cases in the western region of Saudi Arabia in June 2021

Demographics	SARS-CoV-2 Variant			Chi-square Test value	P-value
	Alpha, 11/115 (9.6%)	Beta, 3/115 (2.6%)	Delta, 101/115 (87.8%)		
Gender					
Male	5 (45.5%)	2 (66.7%)	58 (57.4%)	0.71	0.702
Female	6 (54.5%)	1 (33.3%)	43 (42.6%)		
Nationality					
National	7 (63.6%)	2 (66.7%)	91 (90.1%)	7.24	0.027*
Non-National	4 (36.4%)	1 (33.3%)	10 (9.9%)		
Employment					
Unemployed	3 (27.3%)	1 (33.3%)	36 (35.6%)	0.31	0.856
Employed	8 (72.7%)	2 (66.7%)	65 (64.4%)		
Occupation					
Household	1 (12.5%)	1 (50%)	20 (30.8%)	19.69	0.073
Private sector	2 (25%)	0	12 (18.5%)		
Military sector	0	0	14 (21.5%)		
Governmental sector	0	0	4 (6.1%)		
Student	3 (37.5%)	0	5 (7.7%)		
Teacher	1 (12.5%)	0	8 (12.3%)		
Health care worker	1 (12.5%)	1 (50%)	2(3.1%)		

## Discussion

Since the World Health Organization declared the COVID-19 outbreak a pandemic, many SARS-CoV-2 variants have appeared globally. Therefore, our study aimed to detect and explore the distribution and magnitude of the Delta variant in comparison to other variants in June 2021 in the western region of Saudi Arabia.

We found a high incidence of the Delta variant in our study population (n = 101, 87.8%). The Delta variant caused a second wave of COVID-19 cases in Saudi Arabia in December 2021 and was associated with a rise in COVID-19 cases in the United Kingdom (UK), the United States (US), and India [11]. A study in Saudi Arabian COVID-19 patients in April and June 2021 revealed that 40.9% of cases were caused by the Delta variant [1].

We found more males infected with the Delta variant of SARS-CoV-2 than females, which aligned with the proportions of infected males and females in Italy and the US [12]. While the virus can infect people of any age [1], most patients in our study were aged 20 to 30 years, as was seen in other studies in India and the US [13,14]. However, most Saudi population falls within the 20 to 30-year age range [7], which might explain this finding.

Most patients in our study population were vaccinated with at least one dose of vaccine, and we found one breakthrough infection, which was consistent with other studies [15,16]. A study in New York found that 76 people had COVID-19 after receiving two vaccine doses [17]. A UK study enrolled 621 participants and found that 25% of fully vaccinated individuals exposed to the Delta variant via household contact got the disease, compared to 38% of unvaccinated individuals [18].

Of the 115 confirmed cases, 17 had a history of travel. A study in England among 8,500 Delta variant cases found that 29.5% had international travel history [18]. Additionally, 56 people with the Delta variant had contact with a confirmed COVID-19 case, which supports the highly transmissible nature of the Delta variant compared to previous variants [2]. Our study had some important limitations. We could not initiate a case-control study due to a lack of baseline information. Also, the genome sequencing in our study was convenient, not randomized.

## Conclusions

This study aimed to identify the types and distribution of SARS-CoV-2 variants in western Saudi Arabia in June 2021. The high number of cases was primarily due to the Delta variant, which showed higher transmissibility than other variants detected. More genomic sequencing and surveillance are highly recommended to detect and monitor emerging variants. These variants require further understanding of transmission and diagnosis to detect it early and reduce infection by applying the best preventive measures.
